# New strategies for cancer immunotherapy: targeting regulatory T cells

**DOI:** 10.1186/s13073-017-0402-8

**Published:** 2017-01-27

**Authors:** Francesca Finotello, Zlatko Trajanoski

**Affiliations:** Biocenter, Division of Bioinformatics, Medical University of Innsbruck, Innsbruck, 6020 Austria

## Abstract

The immunosuppressive action of regulatory T (T_reg_) cells is one mechanism attributed to the limited success of cancer immunotherapies with checkpoint blockers. Two recent studies report distinct transcriptional profiles of tumor-infiltrating T_reg_ cells and expression of specific molecules, suggesting novel strategies to overcome resistance to cancer immunotherapy.

## Regulatory T cells in cancer immunotherapy

Oncology has been revolutionized by a paradigm shift in the way we treat advanced and/or metastasized cancers. Instead of targeting tumor cells using specific inhibitors, immune cells are targeted to induce or enhance antitumor immunity. Currently, the most promising cancer immunotherapy approach is the blockade of immune checkpoints, which are inhibitory molecules that modulate the amplitude and duration of immune responses [1]. Antibodies that block checkpoint receptors, such as the cytotoxic T-lymphocyte-associated antigen 4 (CTLA4) and programmed cell death protein 1 (PD1), have shown remarkable clinical effects. However, durable responses to checkpoint blockers have been obtained in only a fraction of patients [1], suggesting that persistence of immunosuppressive mechanisms may contribute to intrinsic resistance to cancer immunotherapy.

Cells in the tumor microenvironment attributed with immunosuppressive activities include cancer-associated fibroblasts, myeloid-derived suppressor cells, and CD4^+^ regulatory T (T_reg_) cells expressing the transcription factor FOXP3. Specifically, T_reg_ cells play an essential role in suppressing aberrant immune responses against self-antigens and anti-tumor immune responses. T_reg_ cells are potent suppressors of effector T cells, and, consequently, increased densities of tumor-infiltrating T_reg_ cells have been associated with poor prognosis in a number of cancers [2, 3]. T_reg_ cells can suppress effector T cells through different direct mechanisms, including secretion of inhibitory cytokines, granzyme-mediated cytolysis of effector T cells, and metabolic impairment of effector T cells—for example, by depletion of interleukin-12 (IL-12)—as well as indirect mechanisms such as suppression of dendritic cell maturation and function. However, although widely accepted to be implicated in tumor growth, the molecular phenotypes of tumor-infiltrating T_reg_ cells remain poorly characterized. Identification of molecules expressed specifically in intratumoral T_reg_ cells through genome-wide/transcriptomic analysis has the potential to reveal valuable new targets for therapeutic intervention. Depletion of tumor-infiltrating T_reg_ cells targeting these molecules would shift the balance from immune suppression to immune activation towards tumor cells.

## Unique molecular fingerprints of tumor-infiltrating regulatory T cells

So far, most of the knowledge of these immunosuppressive cells has been derived from the study of peripheral blood T_reg_ cells, whereas a complete portrait of tumor-resident T_reg_ cells, including T_reg_-specific markers, is still lacking. In a recent edition of *Immunity*, two independent studies, led by Rudensky [4] and Pagani [5], shed light on the features of T_reg_ cells infiltrating common human cancers.

The Rudensky laboratory [4] characterized flow-cytometry-sorted T_reg_ cells from human breast carcinomas, peripheral blood, and normal breast parenchyma using RNA sequencing. Tumors with more aggressive phenotypes, such as triple-negative breast cancers (TNBCs), contained higher frequencies of T_reg_ cells, which might imply an active role for T_reg_ cells in cancer progression. RNA-sequencing analysis revealed marked transcriptomic differences between tumor- or tissue-resident T_reg_ versus peripheral blood T_reg_ cells, while the gene expression profiles were highly comparable between T_reg_ cells present in breast tumor and normal tissue. This implies that the tissue of residence, rather than the tumor environment, determines the phenotype of infiltrating T_reg_ cells. The gene expression profiles did, however, reveal an enhanced activation of tumor-infiltrating T_reg_ cells compared to T_reg_ cells present in normal breast parenchyma and the upregulation of genes involved in chemokine signaling and immune cell migration. In particular, the marked expression of chemokine receptor 8 (CCR8), a molecule implicated in the modulation of immunogenicity in colorectal cancer (CRC) [6], was identified as a unique feature of intratumoral T_reg_ cells. This was confirmed in T_reg_ cells isolated from other types of cancers, including CRC, lung cancer, melanoma, and angiosarcoma. Moreover, the authors found a strong negative association between the ratio of CCR8/FOXP3-expressing cells and patient survival.

The Pagani laboratory [5] used RNA sequencing to profile human CD4^+^ T_reg_ cells as well as T helper 1 and T helper 17 cells infiltrating non-small-cell lung cancer (NSCLC), CRC, and the respective matched normal tissues, as well as peripheral blood T_reg_ cells. Their results show a large overlap of the expression profiles from tissue- and tumor-resident T_reg_ cells, yet striking differences with respect to blood T_reg_ cells. Differential expression analysis revealed 309 transcripts consistently upregulated in T_reg_ cells from NSCLC and CRC tumors, but not in other CD4^+^ T cells subpopulations or in tissue-resident T_reg_ cells. The intratumoral signature includes genes associated with increased suppressor activity, such as the immune checkpoints CTLA4, lymphocyte activation gene 3 (LAG3), and T-cell membrane protein 3 (TIM3). Three of the most highly expressed signature genes, layilin (LAYN), MAGE family member H1 (MAGEH1), and CCR8 (the latter two were also identified as a unique feature of tumor-infiltrating T_reg_ cells by the Rudensky laboratory [4]), were associated with a significant worsening of the 5-year survival of both CRC and NSCLC patients. Finally, the authors confirmed the expression signatures shared by CRC- and NSCLC-infiltrating T_reg_ cells via single-cell analysis and demonstrated that they are conserved across different metastatic and non-metastatic cancers.

## Potential for new therapeutic strategies for cancer immunotherapy

The presence of T_reg_ cells in the tumor microenvironment represents one of the main obstacles for successful cancer immunotherapy. Since their immunosuppressive action is exerted through different mechanisms, the selective depletion of tumor-infiltrating T_reg_ cells holds promise for unleashing potent antitumor responses. These two resources [4, 5] now provide the potential means for targeted depletion of T_reg_ cells. Both studies showed that CCR8 is specifically expressed in tumor-infiltrating T_reg_ cells in multiple tumor types, suggesting that CCR8 represents a promising target to selectively deplete T_reg_ immunosuppressive activity in the tumor microenvironment.

The marked differences between tumor-resident and blood-derived T_reg_ cells reported in these studies highlight the importance of assessing the phenotypes of immune cells at the tumor site. The generation of additional transcriptomic data from other tumor-infiltrating immune cells would tremendously benefit the development and optimization of computational approaches for dissecting complex tumor–immune cell interactions [7], and could contribute to the identification of novel mechanistic insights.

A number of questions remain unanswered and should motivate further experimental and clinical studies. For example, the mechanisms underlying the increased infiltration of T_reg_ cells in aggressive cancers such as TNBC, and the implications for cancer prognosis, remain elusive. Furthermore, heterogeneity of tumor-infiltrating T_reg_ cells has not been addressed. It was shown recently that distinct subpopulations of T_reg_ cells contribute in opposing ways to determining CRC prognosis: CRCs infiltrated with suppression-competent T_reg_ cells had a worse prognosis compared with CRCs infiltrated with non-suppressive T_reg_ cells [8]. Thus, additional studies need to be carried out to validate this observation in other cancer types. Finally, it should be noted that the promising candidate for therapeutic targeting, CCR8, is also expressed in cardiomyocytes and liver [9]; thus, straightforward systemic treatment with CCR8 inhibitors might cause serious immune-related adverse effects. Still, the two studies highlight the importance of assessing the phenotypes of immune cells specifically within the tumor site. Further transcriptomic characterization of tumor-infiltrating T_reg_ cells in other cancers, as well as other immunosuppressive cell populations, will be of the utmost importance for the prevention of resistance to cancer immunotherapies.

## Abbreviations

CRC: Colorectal cancer
NSCLC: Non-small-cell lung cancer
TNBC: Triple-negative breast cancer
T_reg_ cell: Regulatory T cell
